# Deleterious KOs in the HLA Class I Antigen Processing and Presentation Machinery Induce Distinct Changes in the Immunopeptidome

**DOI:** 10.1016/j.mcpro.2025.100951

**Published:** 2025-03-18

**Authors:** Ilja E. Shapiro, Clélia Maschke, Justine Michaux, Huisong Pak, Laura Wessling, Tamara Verkerk, Robbert Spaapen, Michal Bassani-Sternberg

**Affiliations:** 1 Department of Oncology, University of Lausanne (UNIL) and Lausanne University Hospital (CHUV), Lausanne, Switzerland; 2 Ludwig Institute for Cancer Research, Lausanne Branch, Lausanne, Switzerland; 3 Agora Cancer Research Centre, Lausanne, Switzerland; 4 Landsteiner Laboratory, University of Amsterdam, Amsterdam, The Netherlands; 5 Department of Immunopathology, Sanquin Research, Amsterdam, The Netherlands

**Keywords:** immunopeptidomics, antigen processing and presentation machinery, human leukocyte antigen class I

## Abstract

The human leukocyte antigen (HLA) processing and presentation machinery (APPM) is altered in various diseases and in response to drug treatments. Defects in the machinery may change presentation levels or alter the repertoire of presented peptides, globally or in an HLA allele–restricted manner, with direct implications for adaptive immunity. In this study, we investigated the immunopeptidome landscape across a panel of isogenic HAP1 cell line clones, each with a KO of a single gene encoding a key protein in the APPM, including B2M, TAP1, TAP2, TAPBP, IRF2, PDIA3, ERAP1, GANAB, SPPL3, CANX, and CALR. We applied immunopeptidomics and proteomics to assess the successful gene KOs on the protein level, understand how these proteins participate in antigen presentation, and contextualize protein expression and antigen presentation. We validated the absence of the KO proteins in the respective samples and found that knocking-out an APPM component leads to the loss of peptide subsets that are normally presented on the control wildtype cells. We assessed the immunopeptidomes qualitatively and quantitatively, considering factors like peptide diversity, peptide length distribution, and binding affinity to the endogenously expressed HLA alleles in HAP1 cells. We demonstrated prominent HLA allele–restricted alterations in several KO conditions. The absence of CALR, CANX, and TAP1 led to significant changes in HLA allele–specific presentation levels. Overall, this work represents the first systematic analysis of how the absence of individual APPM components, knocked out in a single cell line under controlled conditions, affects the immunopeptidome. This approach could facilitate the creation of predictive tools capable of prioritizing HLA-bound peptides likely to be presented when presentation defects occur, such as in cancer and viral infections.

## Highlights

• Altered immunopeptidome in a panel of antigen processing and presentation machinery gene KO cells.
• The impact of a KO on the immunopeptidome reflects a gene's biological context.
• Changes relate to the diversity, origin, and dynamic range of presentation levels of immunopeptides.
• Observed changes are human leukocyte antigen restricted.

## Graphical Abstract

**Figure:**
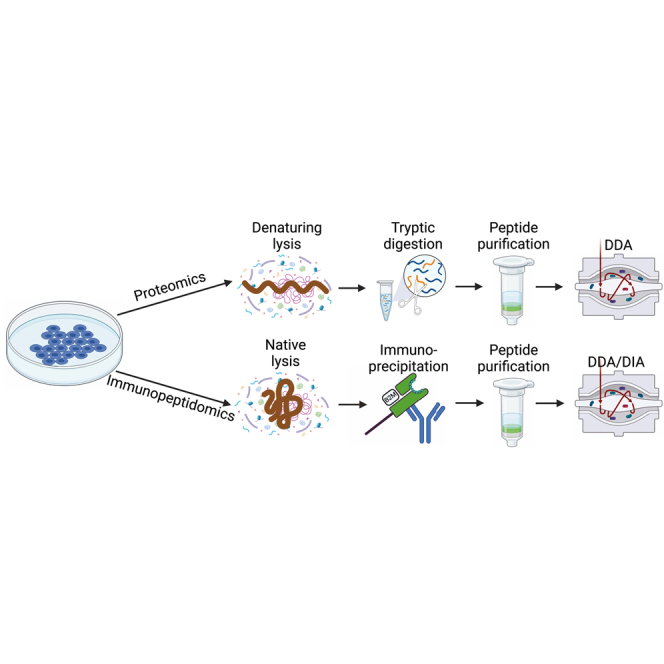

## In Brief

This study explores how knocking out individual components of the HLA processing and presentation machinery in HAP1 cells affects antigen presentation. We demonstrate that the absence of key proteins causes HLA allele–specific changes in peptide presentation, offering insights into immune regulation and potential applications in cancer and viral infections.

Human leukocyte antigen class I (HLA-I) molecules present short peptides, known as immunopeptides, on the cell surface. In cancer, these peptides can arise from intracellular degradation of abnormally expressed, mutated, or pathogenic proteins and are recognized as foreign by cognate T-cell receptors on CD8+ T cells. This recognition triggers the activation of T cells, enabling them to target and eliminate cancer cells (1). The HLA-I antigen processing and presentation machinery (APPM) consists of proteins that function as a molecular assembly line, processing and presenting HLA–peptide complexes on the cell surface. The binding of peptides to HLA molecules is determined by specific interactions between residues of the peptide with the HLA binding groove. Each HLA allotype binds a repertoire of peptides that are compatible with its consensus binding motif (2). APPM members include the HLA protein complex (HLA and B2M), proteins that degrade proteins and polypeptides to short peptides (proteasome, ERAP1, and ERAP2), proteins that are engaged in peptide translocation (*e.g.*, peptide transporter associated with antigen processing; TAP1, TAP2), as well as proteins that belong to the peptide loading complex (PLC) including those responsible for modifying and assembling HLA-I complexes (*e.g.*, GANAB, CANX, PDIA3) and proteins that mount the most suitable peptide onto an HLA complex (TAPBP [tapasin], TAPBPR), a process referred to as peptide editing (2). Additional factors influence functional peptide presentation, including the transcriptional regulator IRF2 and neolacto series glycosphingolipids whose expression is suppressed by SPPL3 (3).

Defects in antigen presentation may change presentation levels or the repertoire of presented peptides, either globally or in an HLA allele–restricted manner. Immune pressure, mediated by elimination of cancer cells by T cells (4), may lead to the selection and expansion of immune-escaping tumor clones carrying mutations in proteins involved in antigen processing and presentation (5). For example, the loss of B2M severely impairs a cell's ability to present HLA-I complexes, which directly affects the responsiveness of cancer patients to immune checkpoint inhibitors (6). In addition, viruses, such as oncoviruses, are masters at evading the immune system by targeting various key cellular mechanisms including the APPM, leading to downregulation of HLA-I from the cell surface (7). For example, in infected CD4+ T cells, the HTLV-1 auxiliary protein p12 interacts with the HLA-I heavy chain, preventing its maturation and resulting in proteasomal degradation of HLA (8). Also, HHV4's BNLF2a protein inhibits CD8+ T-cell recognition of infected cells by inhibiting the ability of TAP to bind peptides and ATP. This inhibition leads to a deficiency of peptide presentation as well as in downregulation of HLA-I (9). Furthermore, drugs targeting the APPM, like ERAP1 inhibitors and interferon gamma, also contribute to reshaping the immunopeptidome by altering peptide editing (10, 11).

Since the APPM is altered in various diseases and in response to drug treatments, there is a growing interest in characterizing the immunopeptidome when the APPM is perturbed. This can improve the prioritization of relevant antigenic peptides for vaccine design, immunomonitoring, and more accurate modeling of T-cell responses. Advanced prediction of antigen presentation requires better understanding of the mechanisms driving the selection of source antigens for presentation and how each protein within the APPM pathway modulates the immunopeptidome (12). However, this requires setting up experimental models where key APPM components are specifically perturbed. In this study, we investigated the immunopeptidome landscape across a panel of APPM KO cells (PAKC). PAKC is a collection of isogenic HAP1 cell line clones, each with a KO of a single gene encoding a key protein in the APPM, including B2M, TAP1, TAP2, TAPBP, IRF2, PDIA3, ERAP1, GANAB, SPPL3, CANX, and CALR. We assessed how these APPM defects influenced HLA-I peptide presentation both qualitatively and quantitatively, considering factors like peptide diversity, peptide length distribution, and binding affinity to the endogenously expressed HLA alleles in HAP1 cells. Our results revealed distinct differences in immunopeptidome composition across the various KOs, with TAP1, TAP2, and TAPBP KOs showing HLA allele–restircted effects. In addition, by integrating shotgun proteomics, we explored the relationship between the cellular proteome and the immunopeptidome, offering insights into the processing and presentation of cellular proteins in these isogenic lines. The systematic analysis performed here shed light on the complexity of the immunopeptidome that is stepwise shaped by the numerous proteins that comprise the APPM. Considering the frequent occurrence of APPM defects, in infectious diseases and in tumors, such information is crucial for understanding how such impairments influence the immunopeptidome and for the further development of disease models.

## Experimental Procedures

### Experimental Design and Statistical Rationale

A detailed description of the immunopeptidomic experimental design, including naming of samples, RAW MS file names, and assignment of biological and technical replicates, is provided in supplemental Table S1. Isogenic cell line samples were expanded and harvested with the same protocol to minimize variation in three biological replicates per condition. To provide maximum immunopeptidome depth, we aimed to measure two technical replicates in data-dependent acquisition (DDA) and two technical replicates in data-independent acquisition (DIA) of all immunopeptide samples. Some measurements could not be processed computationally because of low signal and were hence discarded. In total, 128 immunopeptidomics raw files were suitable for processing.

### Statistical Tests

For comparison between conditions, we applied Student's *t* test. *p-*values were adjusted with the Bonferroni correction method. *p-*values <0.05 were considered significant.

### Reagents

The reagents used for experimental work were as follows: cell culture medium (RPMI1640 GlutaMax-I; Gibco, ref. 61870-010); fetal bovine serum (Gibco, ref. 10437-028); penicillin–streptomycin (catalog no.: 4-01F00-H); PBS (Bichsel, ref. 100 0324); acetonitrile (ACN; Biosolve, UN 1648), formic acid (FA; Thermo Scientific, product code: 85178), iRT peptides (Biognosys, ref. Ki-3002-2), urea (BioChemica, product code: A1360), ammonium bicarbonate (Sigma–Aldrich, ref. A6141), DTT (Fisher Scientific; catalog no.: BP172-25), iodoacetamide (Sigma, ref. I6125-5g), Trypsin/Lys-C Mix (Promega, ref. V5073), and ReproSil-Pur C18 beads (C18 beads; Dr Maisch, ref. r119.aq).

### Cell Culture

HAP1 cell line clones (13, 14) were grown in cell culture medium with 10% of heat-inactivated fetal bovine serum and 100  U/ml penicillin–streptomycin. Following expansion, cells were washed twice in PBS. For immunopeptidomics samples, pellets of 10^8^ cells were stored at −80 °C. For proteomics samples, pellets of 500 × 10^3^ cells were stored at −80 °C.

### Pan-HLA Flow Cytometric Staining on HAP1 Cells

For the analysis of extracellular HLA-I expression, HAP1 KOs were stained with Calcein AM Viability Dye (ThermoFisher Scientific) according to the manufacturer's instructions for discrimination of living cells. Next, staining of HLA–HLA-I complexes was performed using a phycoerythrin-conjugated mouse antihuman HLA-A, -B, and -C antibodies (1:20 dilution, BioLegend), alongside with an appropriate phycoerythrin-conjugated mouse IgG2a, κ isotype control (1:20 dilution, BioLegend) for 15 min at room temperature in the dark. In addition, to the isotype controls, unstained controls were included in the experimental setup. Complimentary to the Calcein-AM staining, a staining with 4',6-diamidino-2-phenylindole (10 mg/ml, 1:200 dilution; Applichem) was included right before measurement for the identification of dead cells. Stained cells were analyzed using the BD FACSymphony A5 (BD Biosciences), and the data were subsequently analyzed with the FlowJo software, version 10.0 (FlowJo LCC). To calculate the HLA-A, -B, and -C surface expression, we deducted the median negative control signal from the median of the median fluorescence intensity distribution of the respective HAP1 cells.

### Immunopeptidomics—Immunoprecipitation

Immunoprecipitation of HLA-I complexes was conducted as described (10). Briefly, we harvested W6/32 antibodies from HB95 hybridoma cells and crosslinked them to Protein A-coated Sepharose beads. After nondenaturing lysis of 10^8^ cells per biological replicate, the cross-linked antibody-covered beads were used to isolate HLA-I complexes using the plate format and the positive pressure manifold. Following washing and elution of beads, eluates were desalted with C18 columns and immunopeptides were eluted with 28% ACN and 0.1% FA, vacuum dried, and stored at −80 °C.

### Immunopeptidomics—Mass Spectrometry Measurement

Vacuum dried peptides were resuspended in 2% ACN and 0.1% FA and spiked with iRT peptides according to the instruction of the supplier. Per sample, we measured four injections, two in DDA and two in DIA. The platform for sample injection, chromatographic separation, and spectral acquisition was an Easy-nLC 1200 coupled to a Q Exactive HF-X (ThermoFisher Scientific). The analytical column used was 450 mm long (8 μm tip, 75 μm ID) and packed with C18 beads. Peptides were separated at a flow rate of 250 nl/min with a gradient of 120 min as described (10) for HLA-I peptides. For DDA measurements, cycles consisted of a mass spectrometry 1 (MS1) scan (scan range = 300–1650 Th, R = 60,000, automatic gain control [AGC] target = 3e6, and maximum injection time [IT] = 80 ms), followed by MS2 scans (R = 30,000, AGC target = 2e5, maximum IT = 120 ms, normalized collision energy = 27, and isolation window = 1.2Th) of isolated precursors according to “Top20” rules, with dynamic exclusion enabled and set to 20 s. For DIA measurements, cycles consisted of an MS1 scan (scan range = 300–1650 Th, R = 120,000, AGC target = 3e6, and maximum IT = 60 ms) and 22 MS scans (window widths = [37, 30, 24, 24, 22, 23, 21, 24, 21, 24, 24, 25, 27, 27,30, 35, 38,43,53, 72, 103, 594] Th, window overlap = 1Th, R = 30,000, AGC target = 3e6, maximum IT = auto, normalized collision energy = stepped [25.5, 27, 30]).

### Immunopeptidomics—Spectral Matching and Peptide Quantification

For library generation from DDA files as well as for searching DIA files with the library, the FragPipe environment (v22.0) was used, with MSFragger and DIA-NN, respectively. The only difference between canonical and noncanonical search was the FASTA file and false discovery rate (FDR) that was used. The FASTA used for the canonical search was the canonical human proteome without isoforms as curated by SwissProt on February 24, 2024, concatenated with iRT peptide entries as well as the list of common contaminants provided by the FragPipe software, with a total of FASTA 20,482 entries. The FASTA used for the noncanonical search was generated by processing previously published HAP1 wildtype transcriptomics data (15) with NeoDisc (16) in fastq mode, applying default parameters to the single-sample pipeline, and adding iRT sequences as well as common contaminants as provided by the FragPipe software. This noncanonical FASTA was based on the GRCh37 assembly with GENCODE v43 annotation, hence, composed of 11 iRT sequences, 118 contaminant sequences, 76,398 canonical (Protein Existence class (PE) = 1), 227,759 noncanonical (PE = 3), 15,142 transposable element (PE = 4), and 12 viral (PE = 5) sequences. Search space settings included peptides between length 8 and 14, with methionine oxidation (UniMod accession #: 35), N-terminal acetylation (UniMod accession #: 1), as well as cysteine carbamidomethylation (UniMod accession #: 4) as variable modifications and no fixed modifications. Protein sequence digestion was set to “unspecific.” The permissible peptide mass range was kept between 200 and 3000. Precursor and fragment mass tolerance was 20 ppm, with “Mass calibration, parameter optimization” turned on. FDRs for the canonical search were all set to 0.01 except for the protein FDR that was set to 1. For the noncanonical search we applied group-specific FDRs set to 0.03 except for the protein FDR that was set to 1, in accordance with a previously reported workflow (17). All other settings were set to default. For library-searching the DIA files with the generated library, we used the DIA-NN module provided in the FragPipe graphical user interface, with the FDR set to 0.01, and we applied the default workflow with mass accuracy to 0 ppm, followed by automated optimization of mass accuracies for MS1 by the first run and for MS2 for each run individually.

### Immunopeptidomics—Data Analysis

Peptides were considered as binders when their predicted binding affinity %-rank lied below 2 (18). To calculate the quantification of identified peptides, we summed the maximum value of each single precursor derived from a single peptide together, as advised by the software developers. Intensities used for peptides identified in a sample corresponded to the average of said peptide's quantification across technical replicates. For the immunopeptidomics-based upregulation analysis, we imputed values of absent peptides to be a random number between 1 and 2. Sampling density scores were calculated as described (19, 20, 21). Briefly the sampling density score for an example protein *p* was calculated over several steps. First, each peptide that mapped back to *p* was given a score, calculated by having a standard score, which was reduced slightly for each protein in the database the peptide maps to ($f\left(p\right)=1+0.8^{n}$), where *p* is a peptide and *n* are the number of proteins it maps to. The purpose of lowering the peptide's score was to punish the eventual sampling score of nonproteotypic peptides. The scores of all peptides that mapped to protein *p* were then summed up. This sum was then normalized over the number of theoretical 9-mers that can be derived from that protein ($f\left(P\right)=\frac{\sum_{i=1}^{j}f\left(p_{i}\right)}{L\left(P\right)-8}$, where *p* is a protein, *j* are the number of peptides that map to *p*, and L(*p*) is the protein's length. Binding affinity predictions for each peptide were conducted with MixMHCpred (version 2.2) (18). Data analysis was conducted with the programming languages Julia and R. Plots were generated with the ggplot2 environment in R. For Gene Ontology (GO) analyses, we used the R package ClusterProfileR (22), with all settings set to default apart from the background genes, which are indicated in the respective analysis.

### Proteomics—Sample Preparation for Shotgun Proteomics

Sample preparation followed the same procedure as described (23). Briefly, 500 × 10^3^ cells per sample were lysed and denatured in 8 M urea, 50 mM ammonium bicarbonate, reduced with 5 mM DTT, alkylated with 15 mM iodoacetamide, and digested with Trypsin/Lys-C Mix. Peptides were then desalted in C18 spin columns, dissociated, and stored at −80 °C.

### Proteomics—MS Measurement

Vacuum dried peptides were resuspended in 2% ACN, 0.1% FA, and spiked with iRT peptides according to the instruction of the supplier. Per sample, we measured one DDA injection. The platform for sample injection, chromatographic separation, and spectral acquisition was an Easy-nLC 1200 coupled to a Q Exactive HF-X. The analytical column used was 450 mm long (8 μm tip, 75 μm ID) and packed with C18 beads. Peptides were separated at a flow rate of 250 nl/min with gradients and acquisition parameters as described (23).

### Proteomics—Spectral Matching and Peptide Quantification

DDA files were searched with MSFragger in the FragPipe environment (v22.0). The FASTA used was the canonical human proteome without isoforms as curated by SwissProt on February 24, 2024, concatenated with iRT peptide entries as well as the list of common contaminants provided by the FragPipe software, with a total of FASTA 20,482 entries. All settings were kept as provided with by “Default” workflow, including MS1-based quantification with help of the IonQuant module. A maximum of two missed cleavages were permitted. Precursor and fragment mass tolerances were set to 20 ppm, with “Mass calibration, parameter optimization” turned on. Cysteine carbamidomethylation was set as fixed modifications, and methionine oxidation and N-terminal acetylation were set as variable modifications. Protein and peptide FDR were set to 0.01.

### Proteomics—Data Analysis

Data analysis was conducted with Julia, for computationally demanding analyses, and R. Proteins that were identified with only one peptide were removed from the analysis. Plots were generated with ggplot2, as well as further supplementary packages, with R.

### Data Analysis—Overlap Coefficients

The overlap coefficient is a metric that assesses the similarity of two sets by calculating the fraction of elements found in the smaller set that are also found in the larger set: $overlap\left(A,B\right)=\frac{\left|A\cap B\right|}{\min\left(\left|A\right|,\left|B\right|\right)}$. It is a helpful tool to compare pools of identified peptides or proteins from two samples while normalizing for differences in absolute number of identifications.

### Data Analysis—Intensity Rank Scores

To derive intensity rank scores, we assigned a rank to each peptide identified in a sample from most to least abundant peptide, the most intense peptide being rank 1. The intensity rank score within a sample was calculated with the formula $f\left(x\right)=1-\frac{rank\left(x\right)}{tnp}$, where *tnp* represents the total number of peptides identified in a sample. As such, the least abundant peptide had a rank intensity score of 0, the closer a peptide's rank intensity was to 1 the more abundant it was.

## Results

### In-Depth Immunopeptidomics

**Fig. 1:**
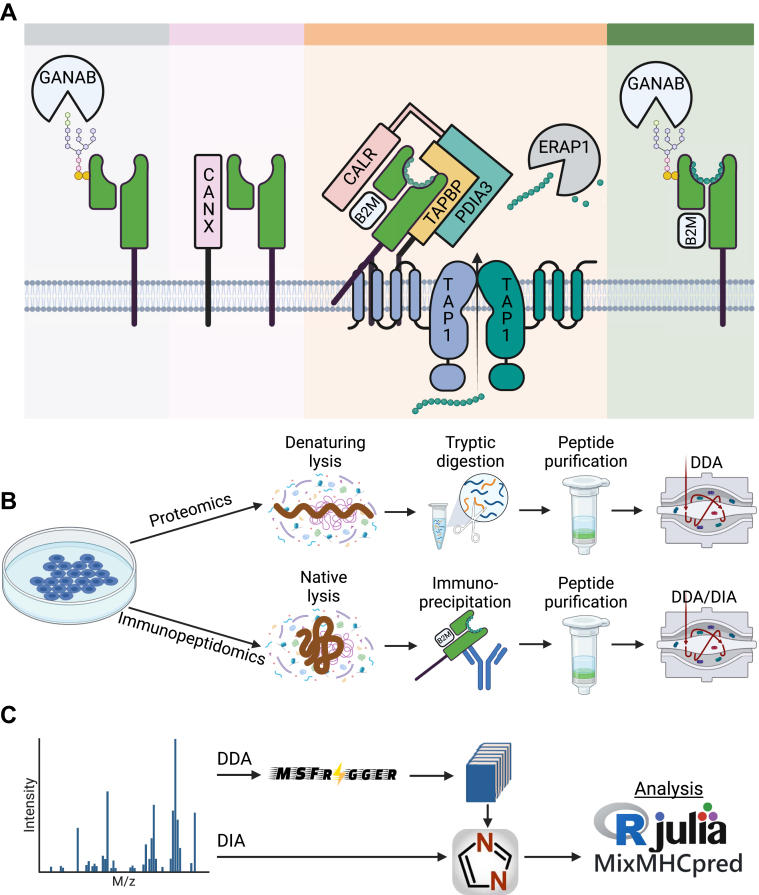
**Proteomics and immunopeptidomics investigation of the antigen processing and presentation machinery.***A*, investigated mediators of HLA modification (GANAB), folding (CANX, CALR), assembly (CANX, CALR, PDIA3), loading (CALR, PDIA3, TAPBP, TAP1, TAP2), editing (TAPBP), and translocation (GANAB). *B*, sample processing for proteomics and immunopeptidomics. *C*, tools used for data processing and analysis. HLA, human leukocyte antigen.

To characterize how various key APPM proteins impact the immunopeptidome (Fig. 1*A*), we performed deep quantitative and qualitative analyses of the immunopeptidome and proteome of an established panel of 11 isogenic HAP1 cell lines, each having an individual KO gene, and compared with the wildtype cells (12 isogenic cell lines in total) (Fig. 1*B*). HAP1 cells are particularly suitable for this purpose; thanks to their near-haploid genome and the expression of the common HLA-A∗02:01, HLA-B∗40:01, and HLA-C∗03:04 (24). From each KO condition, 100 million cells were expanded in triplicates and subjected to immunoaffinity purification of HLA-I complexes. Bound peptides were extracted, purified, and measured on a Q Exactive HFX with DDA and DIA methods, respectively (supplemental Table S1). Furthermore, total protein content was extracted from the cells for standard shotgun proteomics analyses. The acquired MS data were processed with the FragPipe environment (Fig. 1*C*).

**Fig. 2:**
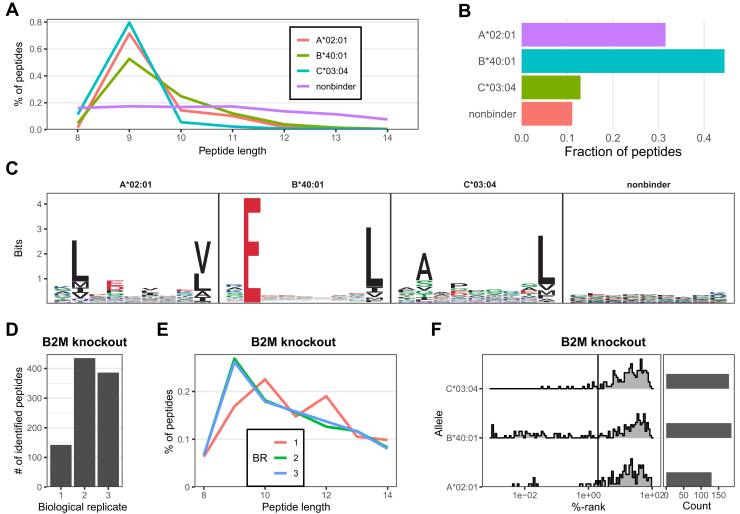
**Library generation for high-sensitivity immunopeptidomics.***A*, HLA allele–wise length distribution of peptides in the spectral library (n = 23,069). *B*, predicted HLA allele proportions of bound peptides in the spectral library (n = 23,069). *C*, motifs of 9-mer peptides in the library with their associated HLA allele (n = 7027). *D*, number of peptide identifications in B2M KO samples. *E*, length distribution of peptides identified in B2M KO samples (n = 497). *F*, binding affinity %-rank of peptides detected in B2M KO samples. HLA, human leukocyte antigen.

To achieve maximum depth, we employed a strategy in which we constructed a comprehensive library from DDA measurements of all conditions and subsequently used this library to analyze the DIA data. The library contained a total of 23,069 unique peptide sequences derived from 8345 source proteins. The peptides exhibited the expected length distribution for HLA-I peptides (Fig. 2*A*). By calculating the predicted binding affinity of the peptides to the three HLA allotypes of HAP1 cells, we assigned each peptide to one HLA-I allotype based on the best predicted affinity. About 89% of the 8 to 14 mer peptides in the library were predicted to bind with rank ≤2% to at least one of the three HLA alleles of HAP1 cells (Fig. 2*B*). As elaborated later, this fraction of binders is lower than expected and related to detection of background impurities in the B2M KO samples. Sequence motifs of the 9-mer peptides predicted to bind the HLAs show the expected binding motifs in reference to the MHCmotifAtlas (Fig. 2*C*) (25).

B2M is indispensable for HLA-I presentation. Immunopeptidomics of B2M KO cells led to the detection of only 497 peptides, comprising 2.3% of the identified peptides in this dataset (Fig. 2*D*). The length distribution of the peptides found in the B2M KO samples deviated substantially from expected distributions (Fig. 2*E*). Likewise, the predicted binding affinity distribution for all biological replicates of the B2M KO cell line revealed few to no binders (Fig. 2*F*). Hence, we considered the B2M KO cell line as a negative control and the peptides detected in B2M KO samples as impurities. We therefore removed these peptides from the subsequent analyses of the remaining 11 isogenic lines.

### Differences in Diversity of the Immunopeptidome in HAP1 APPM KO Cells

**Fig. 3:**
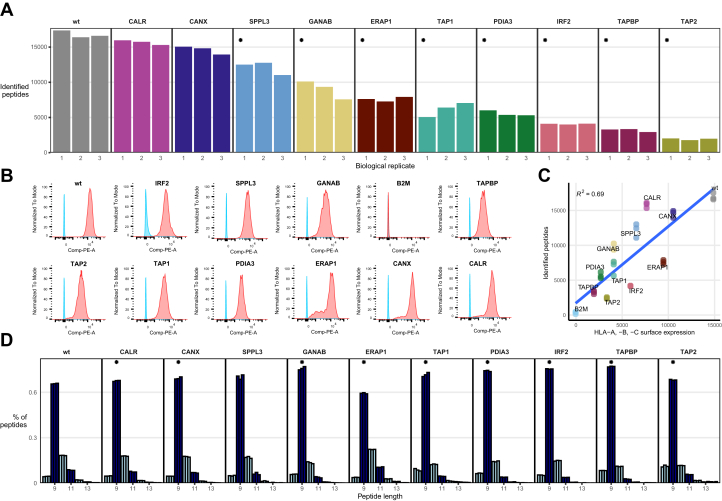
**Immunopeptidome diversity and HLA surface expression.***A*, immunopeptidome diversity, measured by the number of different peptides identified per replicate. *B*, the median fluorescence intensity (MFI) distribution of negative control (*blue*) and cells (*orange*). *C*, linear regression calculating the correlation between HLA-A, -B, -C surface expression and immunopeptidome diversity (*R*^2^ = 0.69). *D*, length distribution of immunopeptidome per sample. *Asterisks* indicate KO conditions with significantly different fraction of 9-mers than observed in wildtype samples. HLA, human leukocyte antigen.

The impact of the various KOs became evident when comparing immunopeptidome diversity, reflected by the number of peptides identified per sample, across different conditions (Fig. 3*A*). Overall, the wildtype samples showed the highest peptide diversity, with 16,394 to 17,365 unique peptide sequences identified. Conversely, the lowest peptide diversity was found in the TAP2 KO, followed by TAPBP, IRF2, TAP1, and PDIA3 KOs. ERAP1, GANAB, and SPPL3 KO conditions demonstrated an intermediate diversity, whereas CANX and CALR KOs had minimal impact on peptide diversity, compared with the wildtype cells. We found statistically significant differences in peptide diversity between KO and wildtype for all conditions except for CANX and CALR (Fig. 3*A*). In general, the APPM is sensitive to KOs regarding the presentable immunopeptidome, with TAP1, TAP2, IRF2, TAPBP, PDIA, GANAB, and ERAP1 KOs resulting in an immunopeptide diversity reduction by over 50%. Indeed, cell surface HLA-I expression, as determined by fluorescence-activated cell sorting analysis of cells labeled with pan-HLA-I antibody (Fig. 3*B*), correlated positively with the immunopeptidome diversity (*R*^2^ = 0.69, Fig. 3*C*).

TAP1 and TAP2 together make up the heterodimer TAP that is a crucial part of the PLC, contributing to the PLC structural integrity by being in direct association with TAPBP (Fig. 1*A*). In addition, TAP facilitates the peptide translocation from the cytosol to the endoplasmic reticulum (ER) lumen after source proteins have been degraded in the cytosol (26). KO of TAP has been shown to promote tumor growth *in vivo* (27). TAP1 and TAP2 KOs present substantially different immunopeptidomes (*p* = 0.0174): though both KOs reduced immunopeptide diversity significantly compared with the wildtype samples, TAP1 at 57 to 71% and TAP2 at 88 to 90%, TAP2 KO showed 60 to 75% less immunopeptide diversity than TAP1 (Fig. 3*A*).

IRF2 is generally known as a transcriptional repressor. Kriegsman *et al*. (28) found that IRF2 was a transcriptional activator of many key components of the HLA-I pathway, including immunoproteasomes, TAP and ERAP1. They demonstrated that upon loss of IRF2, cytosol-to-ER peptide transport and N-terminal peptide trimming become rate limiting for antigen presentation. Indeed, our immunopeptidomic analysis revealed that KO of the IRF2 transcription factor in HAP1 cells significantly decreased immunopeptidome diversity by 75 to 77% (*p* = 0.004), demonstrating the striking role IRF2 has on regulating HLA presentation.

GANAB cleaves off glucose residues of glycans and is involved in the APPM by trimming HLA-I glycans on two levels: first, in order to create a binding site for downstream chaperones to modify the HLA-I complex in the PLC, and second, to enable the translocation of the loaded HLA-I complex from the ER to the cell surface (29, 30). We find a significant reduction in immunopeptidome diversity, ranging from 38 to 54%, following the GANAB KO (*p* = 0.04, Fig. 3*A*).

In all conditions except for SPPL3, we observed statistically significant shifts in average peptide length compared with the wildtype. This indicates differential changes in peptide length preference, depending on the type of APPM perturbation (Fig. 3*D*). ERAP1 is the only gene in the PAKC that directly affects peptide length through its enzymatic activity. It is an aminopeptidase localized in vicinity of the PLC in the ER lumen, capable of trimming the N-terminus of peptides prior and possibly after their loading on the HLA (31, 32, 33, 34). As such, each trimming event of ERAP1 can potentially add a new peptide to the immunopeptidome repertoire. We observed the expected significant increase in the fraction of longer peptides for ERAP1 KOs (*p* = 0.0013). All other KO conditions revealed a decrease in peptide length. To conclude, changes in peptide length do not mirror changes in immunopeptidome diversity, indicating orthogonality between these two characteristics upon APPM component KO.

### The Immunopeptidome of KO Conditions is a Subset of the Wildtype Immunopeptidome

**Fig. 4:**
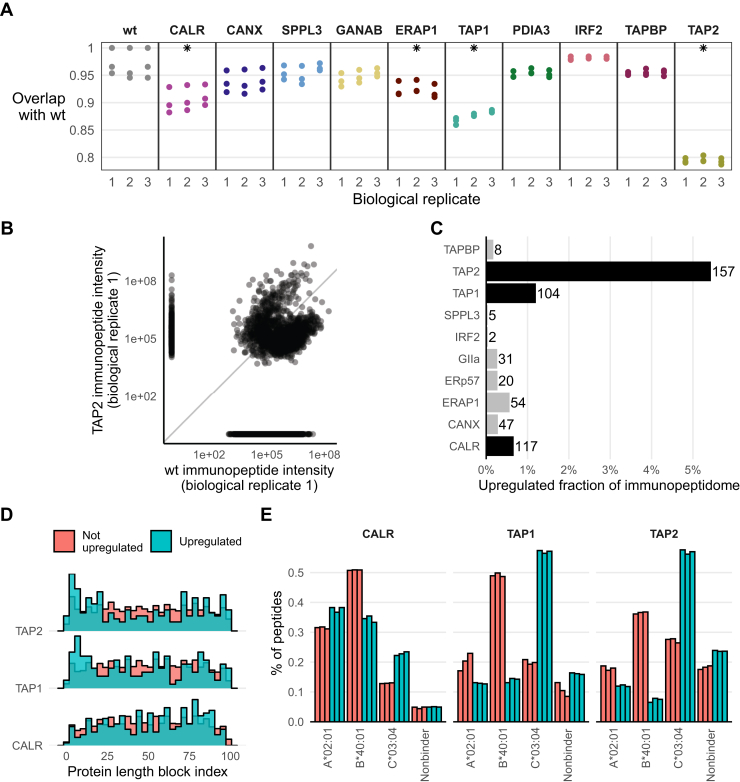
**Immunopeptidome subsets in KOs and upregulated immunopeptidome presentation.***A*, overlap coefficients of individual replicates with all three wildtype replicates. *Asterisks* indicate KO conditions with significantly different overlap coefficients than observed within wildtype samples. *B*, example of intensity, that is, presentation level, comparison between the immunopeptidome of a TAP2 and a wildtype biological replicate. The *dots* cast to either the right side or the bottom were not identified in one of the samples. *C*, number (spelled out next to bar) and fraction (*x*-axis) of upregulated immunopeptides as derived with paired *t* tests (Bonferroni-corrected *p* value ≤0.05, fold change >2). *D*, mapping of upregulated (*blue*) or nonupregulated (*red*) immunopeptides to protein blocks 1 to 100 (*x*-axis) of respective source proteins. *E*, proportion of peptides that bind to a given HAP1 HLA allele (allele distribution) of upregulated (*blue*) or nonupregulated (*red*) immunopeptides. HLA, human leukocyte antigen.

Next, we assessed if the KO conditions present a unique pool of peptides. First, we demonstrated that the immunopeptidome diversity between replicates of all conditions was highly reproducible. For example, among all peptides identified in all three biological replicates of the wildtype cell line, 74% have a coefficient of variation of 0.3 or less in their measured intensity, suggesting good reproducibility in their detection (supplemental Fig. S1*A*). Next, we assessed the similarity between conditions by calculating the overlap coefficients of their peptide populations (Fig. 4*A*). All conditions bear overlap coefficients between 78% and 98% with the three wildtype replicates. The overlap coefficients of wildtype samples with themselves range between 95% and 97% (Fig. 4*A*). This suggests that the general effects seen by APPM KOs on immunopeptidome diversity appears more as reduction of the wildtype repertoire and less as a shift toward a new pool of peptides.

Nevertheless, we find statistically significant differences in overlap coefficient between KO and wildtype for the conditions TAP1, TAP2, CALR, and ERAP1, suggesting that these APPM proteins play a crucial role in shaping the immunopeptidome repertoire (Fig. 4*A*). For example, peptide intensity correlation of peptides detected in TAP2 KO and wildtype conditions revealed a subset of peptides more abundantly presented in the TAP2 KO cells (Fig. 4*B* and supplemental Fig. S2). A differential abundance analysis revealed that despite reduced immunopeptidome diversity, there are populations of immunopeptides with upregulated presentation levels compared with the wildtype (Fig. 4*C*). CALR, TAP1, and TAP2 KOs upregulated the presentation of 100 or more peptides (*p* ≤ 0.05, fold change ≥2), which represents a fraction of 0.7%, 1.2%, and 5.4% of their respective immunopeptidome diversity (Fig. 4*C*). In contrast to CALR, upregulated peptides in the TAP1 and TAP2 KO cells were sampled with a bias toward the source protein's N terminus (Fig. 4*D*), where signal peptides are typically located (16). Interestingly, upregulated peptides showed distinct allele distributions (Fig. 4*E*), underscoring a differential effect of perturbations on HLA alleles. A GO enrichment analysis of source proteins of upregulated peptides showed affected genes are enriched for metabolic processing, structural function, membranal proteins for CALR, TAP2, and TAP2, respectively (supplemental Fig. S3).

### Presentation Across Conditions in Relation to Presentation Level and Source Protein Sampling

**Fig. 5:**
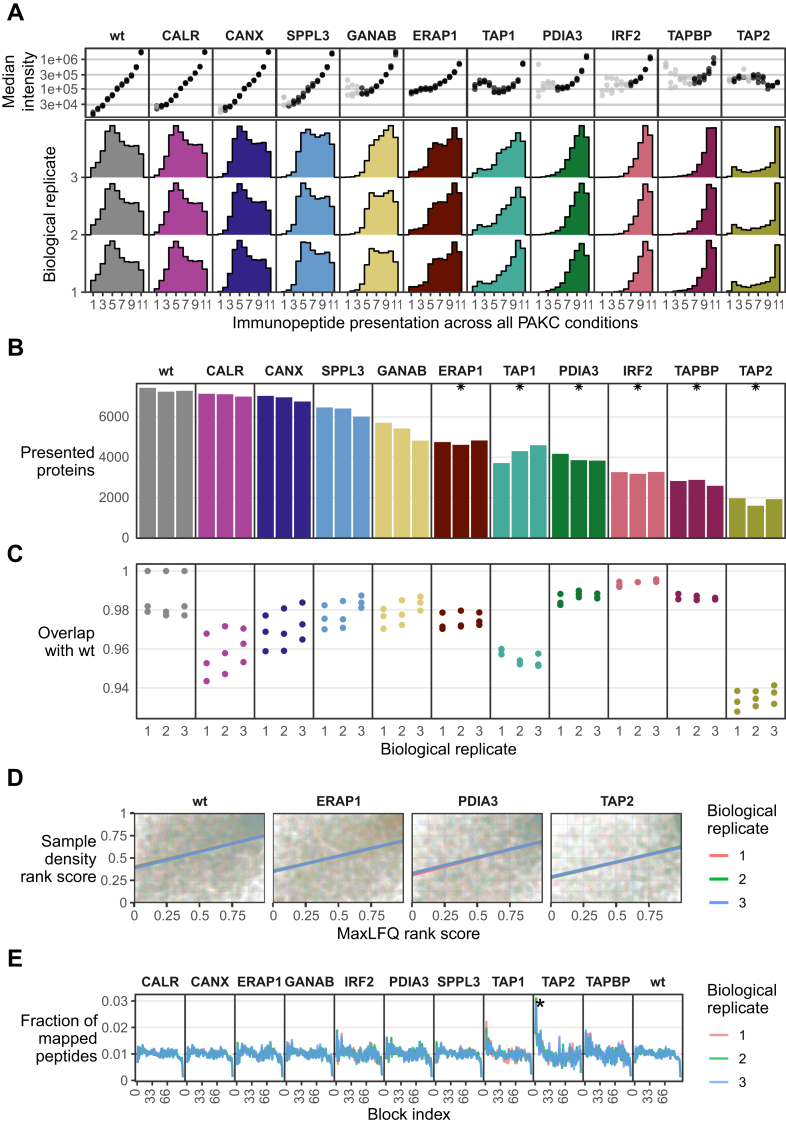
**Immunopeptidome sampling from proteome.***A*, for each sample, the histograms show in how many other conditions the identified immunopeptides were found. The side plot above shows the median intensity for all peptides in a histogram bin, per sample. *Gray dots* indicate a peptide population below 100. *B*, number of proteins per sample that are presented in the immunopeptidome. *C*, overlap coefficients of proteins that are presented in the immunopeptidome. Overlaps are calculated between each sample and the three wildtype replicates. *D*, regression of relative protein intensity (MaxLFQ rank score) and the relative protein sampling density (sample density rank score). Only proteins that were identified as expressed in the proteomics data and as presented in the immunopeptidomics data were included in the analysis. *E*, fraction of all immunopeptides detected (*y*-axis) mapping to protein blocks 1 to 100 (*x*-axis), per replicate. *Asterisks* indicate Bonferroni-corrected *p* values <0.05 when comparing a respective KO condition to the wildtype.

Our results show that the presented immunopeptidome in APPM KOs is primarily a subset of the wildtype immunopeptidome (Fig. 4*A*). To further explore differences in the immunopeptidome across conditions, we examined the number of conditions in which peptides are detected and at what abundances. Interestingly, the intensity of peptides in the wildtype, CALR, and CALN KO cells decreases strictly the fewer other conditions these peptides occur in (Fig. 5*A*). This trend is severely overthrown for TAP1 and TAP2 KOs, whereas ERAP1, GANAB, and PDIA3 KOs also deviate by flattening the dynamic range of presented peptides (Fig. 5*A*). The narrower dynamic range of GANAB, IRF2, PDIA3, and TAPBP KOs is likely related to perturbations in peptide editing and loading, whereas for TAP1 and TAP2 KOs, the dysfunctional peptide translocation into the ER is limiting the available peptide pool. ERAP1 KO also changes the available peptide pool, whereby the decrease in lowly presented peptides might be due to the limited presentation of peptides that are fully depend on ERAP1 activity. The two factors at play here are the available peptide pool and the capacity for peptide loading and editing, and our data suggest that a functional APPM maintains a degree of immunopeptidome diversity as well as dynamic range of presentation levels.

To gain a better understanding of protein expression and the sampling of proteins for HLA presentation, we generated proteomics data from bulk tryptic digests of each individual KO cell line (Fig. 1*B*). Proteins from genes that were knocked-out in different clones were absent in the respective samples (supplemental Fig. S4*A*). Hence, the proteomic analysis confirmed the successful KO of APPM genes. As expected, the number of presented proteins agrees with the total number of identified peptides per sample (Figs. 3*A* and 5*B*). In line with findings at the peptide level, proteins presented in KOs overwhelmingly consist of subsets of proteins presented by the wildtype cells (Fig. 5*C*). Significant GO enrichment terms of proteins that are not presented in KOs compared with the wildtype are scarce. We observe patterns in few instances, for example that particularly transmembrane proteins are absent in ERAP1 KO cells (supplemental Fig. S4*B*).

**Sup fig 5:**
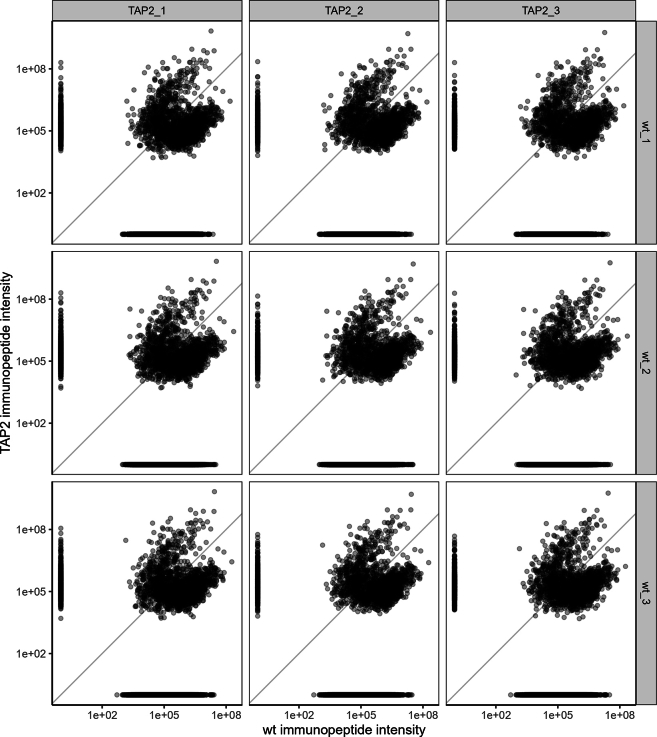


To compare the relation between protein expression and protein presentability, we examined protein expression and calculated the protein sampling density for each protein (21) (supplemental Fig. S5, *A* and *B*). As expected, proteins with higher expression levels are also more presented, and this was consistent across all KO conditions, as exemplified for the ERAP1, PDIA3, and TAP2 KOs and the wildtype (Fig. 5*D*). Next, we explored potential differences in the processing of source proteins, related to the sampling of peptides along the protein sequence. First, we divided all protein sequences into 100 equidistant blocks to normalize protein lengths and the associated positioning of derived immunopeptides. Next, by identifying in what block the N-terminus of each immunopeptides lies, we identified presented regions across proteins overall. We allocated immunopeptides by their N-terminus to map them to a single amino acid coordinate on a protein, so that each immunopeptide can be placed in a single length block. Globally, immunopeptides originated from all possible positions from the N- to the C-terminal regions of proteins (Fig. 5*E*). Preferential presentation in the N-terminus significantly stands out, as it is the most well presented in IRF2, PDIA3, TAP1, TAP2, and TAPBP KO conditions. This observation is most pronounced for TAP1, TAP2, and TAPBP KOs, where N-enriched peptides represent 2%, 3%, and 2% of immunopeptides, respectively (Fig. 5*E*). Statistically, N-terminus enrichment was only significant for TAP2 (*p* = 0.0497), in agreement with the preferential presentation analysis described previously (Fig. 4*E*). Importantly, the drop in presentation at the protein C-terminus reflects a computational artifact, as peptides were assigned based on the location of their N-terminus. Therefore, at a minimal peptide length of 8, the N-terminal position cannot come closer than eight amino acids away from the protein C-terminus.

### Qualitative and Quantitative Differences in the Immunopeptidome are HLA Restricted

**Fig. 6:**
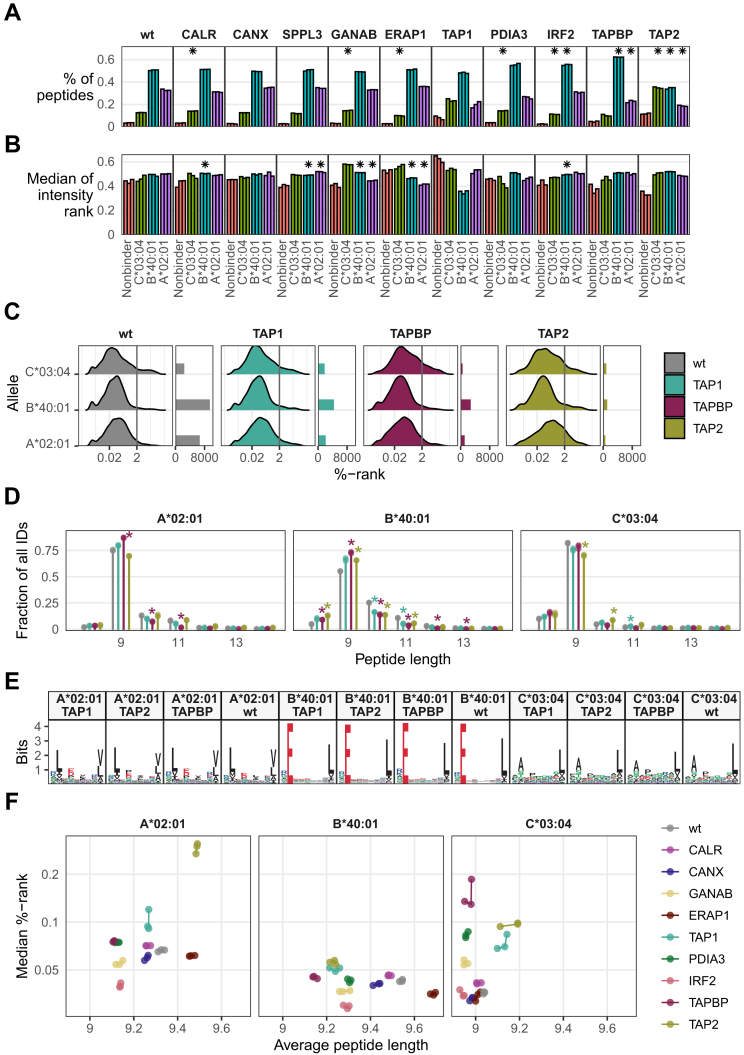
**APPM KO consequences are HLA restricted.***A*, proportion of peptides that bind to a given HAP1 HLA allele (allele distribution), per condition. *Asterisks* indicate HLA allele fractions that deviate significantly from the allele fraction observed in the wildtype. Nonbinder fractions were not evaluated. *B*, median of intensity rank score of all peptides bound by a given HLA allele, per condition. *Asterisks* indicate HLA allele–wise peptide intensity rank scores that deviate significantly from the intensity rank scores observed in the wildtype. Nonbinder intensities were not evaluated. *C*, %-rank distribution of peptides detected in TAP1, TAP2, TAPBP KO clones, as well as in wildtype HAP1 cells. Side plot indicates the absolute number of peptides depicted in distribution. *Vertical*, *dark gray line* indicates the typical cutoff for immunopeptidomics experiments to assign peptides as binders and nonbinders. *D*, length distribution of peptides identified in TAP1, TAP2, TAPBP KO clones, as well as in wildtype HAP1 cells. Peptide populations are divided by the HLA allele peptides bind. *Asterisks* indicate statistically significant (*p* ≤ 0.05, Student's *t* test, Bonferroni-corrected) differences between condition and wildtype for each peptide length, per HLA allele. *E*, motif of 9-mers in TAP1, TAP2, TAPBP KO clones, as well as in wildtype HAP1 cells, divided by which HLA allele peptides bind. *Asterisks* indicate Bonferroni-corrected *p* values <0.05 when comparing a respective KO condition to the wildtype. *F*, average peptide length as well as median %-rank of peptides found in individual samples (each dot a sample), peptide populations divided by which HLA allele peptides bind. *Lines* connecting samples indicate groups of replicates. APPM, antigen processing and presentation machinery; HLA, human leukocyte antigen.

In wildtype samples, ∼33% of peptides are predicted to bind to HLA-A∗02:01, ∼50% to HLA-B∗40:01, ∼12% to HLA-C∗03:04, and 4% of identified peptides are considered nonbinders (Fig. 6*A*). TAP2 is the only KO condition that significantly deviates in its respective fraction of binders for all three HLA alleles. Changes in two of three HLA alleles were found for IRF2 and TAPBP, where only B∗40:01 and C∗03:04 or A∗02:01 and C∗03:04 remained unchanged, respectively. In CALR, ERAP1, PDIA3, and GANAB KOs, only C∗03:04 had a significantly deviant fraction of binders compared with the wildtype. No changes of statistical significance were observed in CANX, SPPL3, and TAP1 KOs. Although the results do not reach statistical significance, because of the reduced number of identified peptides, there are notable findings for the TAP1 KO. Between 16% and 23% of the peptides are predicted to bind to the A∗02:01 allele, compared with 32% in the wildtype. In contrast, 23% of peptides are predicted to bind to the C∗03:04 allele in the TAP1 KO, compared with 13% in the wildtype.

**Fig. 7:**
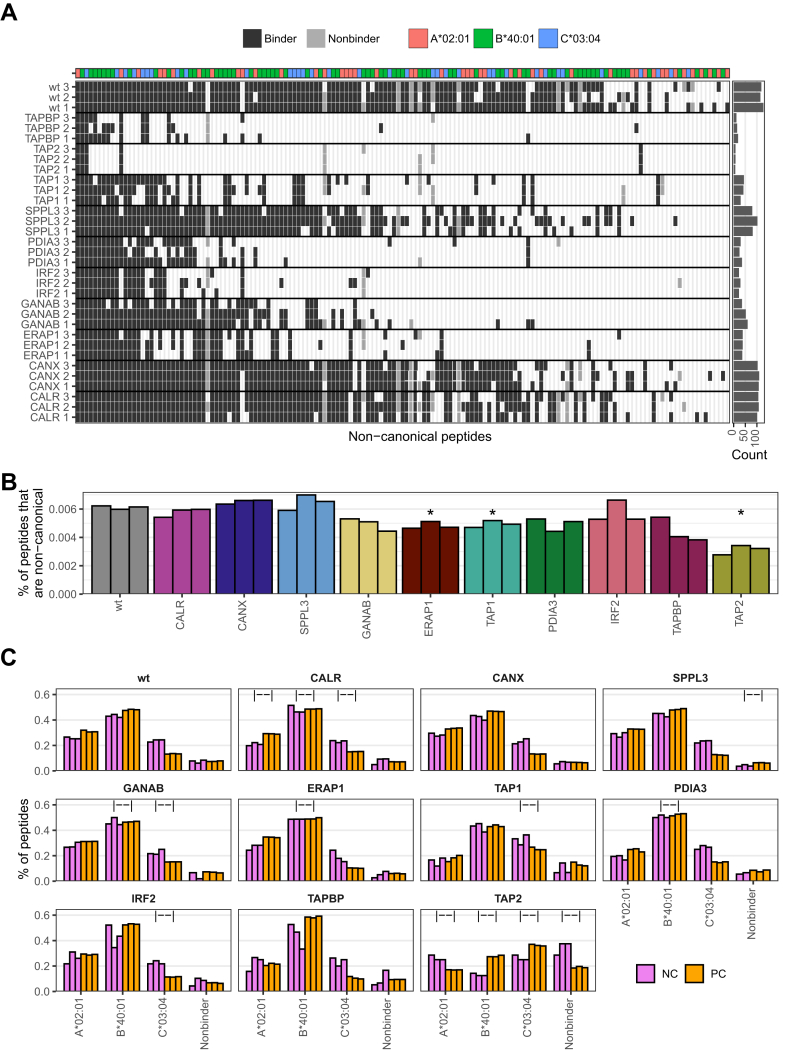
**Noncanonical (NC) immunopeptides populate the PAKC immunopeptidome.***A*, tile plot where each column represents a unique NC immunopeptide and tiles indicate that said immunopeptide was detected in the sample indicated on the *y*-axis. *Dark tiles* indicate a %-rank <2 for at least one of the HAP1 HLA I alleles (binder). *Top-side plot* indicates to what classical HLA I alleles the peptide is predicted to bind to best, the *right-side plot* indicates the total number of NC immunopeptides identified per sample. *B*, fraction of NC immunopeptides across the entire immunopeptidome per sample. *Asterisks* indicate Bonferroni-corrected *p* values <0.05 when comparing a respective KO condition to the wildtype. *C*, differences in allele distribution between immunopeptides derived from canonical (PC) and NC peptides. “|--|” indicates Bonferroni-corrected *p* values <0.05 when comparing fractions of PC and NC immunopeptides for a given allele within a condition. HLA, human leukocyte antigen; PAKC, panel of APPM KO cell.

Next, we explored quantitative differences in HLA allele binding (Fig. 6*B*). For this, we calculated the intensity rank distribution of each HLA allele, an estimation of their presentation levels based on the intensity of the associated peptides. In the wildtype, the intensity rank distribution of all three HLA alleles is centered at almost equal levels according to the median, with HLA-A-binder presentation levels lying 3 to 4% lower than HLA-B- and HLA-C-binders. For 3 of 10 KOs, CALR, CANX, and TAP1, HLA allele–related significant change in presentation level was found for A∗02:01 as well as B∗40:01. Interestingly, in ERAP1 and GANAB KOs, only B∗40:01 presentation levels changed significantly. Furthermore, while the HLA allele binding composition of CALR looks similar to the wildtype, the surface abundance of individual binders of each HLA allele deviates strongly from those observed in the wildtype.

To exemplify more subtle changes in the immunopeptidome, we compared TAP1, TAP2, and TAPBP KO conditions for their allele-specific changes. Beyond macroscopic shift in how many peptides are associated with each HLA allele, closer inspection of these conditions' %-rank distribution of each HLA allele–allocated population of immunopeptides revealed shifts in the overall binding affinity (Fig. 6*C*). For TAP1, TAP2, and TAPBP KOs, we found a strong shift in the HLA allele–binding composition of the immunopeptidome. Examination of the %-rank distribution of peptides assigned to each HLA allele revealed also an overall shift in peptide binding affinity compared with the wildtype. While affinity distributions for A∗02:01-binders remained unchanged for TAP1 and TAPBP, we saw a clear shift to weaker binding peptides for TAP2. This is accompanied by slight shifts to weaker binding peptides for C∗03:04-binders, which are also observable for TAPBP. For B∗40:01-binders, we do not observe changing %-rank distributions for neither TAP1, TAP2, nor TAPBP.

We also inspected changes in peptide length preference of different HLA alleles in TAP1, TAP2, and TAPBP KO models (Fig. 6*D*). B∗40:01-binders, despite retaining their %-rank distribution, show clear deviations in length preference compared with the wildtype, with statistically significant increased presentation of 8- and 9-mers for TAP2 and TAPBP, and significant decrease in the fraction of 10- and 11-mers for TAP1, TAP2, and TAPBP KO conditions. For A∗02:01-binders, TAP1 and TAP2 KOs showed a similar length distribution as the wildtype, whereas TAPBP KOs resulted in a significant increase in the fraction of 9-mers, whereas 10- and 11-mers were significantly decreased. We also wanted to compare whether the sequence motifs of HLA allele–binding peptide population looked different when either TAP1, TAP2, or TAPB was knocked-out. By randomly sampling 400 predicted binders per HLA allele per condition, we demonstrated that the motifs of 9-mers of each set of HLA allele-binders for TAP1, TAP2, and TAPBP do not deviate from the motifs seen for the wildtype. This, despite the differences determined previously (Fig. 6*E*).

The observation that knocking-out components of APPM affect peptide length preference and binding affinity of bound peptides in an HLA allele–specific fashion can be extended to all other KOs as well (Fig. 6*F*). While for B∗40:01 APPM KOs affect peptide length more than peptide binding affinity, for C∗03:04 the opposite is the case. Meanwhile, for A∗02:01, we found pronounced changes for the different KOs in both dimensions. Of note, shifts in %-rank distribution of peptides, when comparing KOs to the wildtype, indicate a higher tolerance of the APPM to present peptides with a poorer binding affinity, possibly driven by a change in peptide availability. This highlights the biological impact upon the absence of an APPM component.

### KO of APPM Genes Can Restrict Access to Immunopeptides from Noncanonical Sources

To understand whether the KO of APPM genes changes the presentation of noncanonical peptides, we searched the immunopeptidome data against a reference database comprising canonical and noncanonical sequences (*e.g.*, long noncoding RNAs and pseudogenes), found to be expressed in HAP1 transcriptomics data (15), applying group-specific FDR thresholds of 3% (17). In total, excluding the B2M KO, we identified between 9 and 130 noncanonical sequences, representing 151 unique peptides in total (Fig. 7*A*). Like for canonical peptides, APPM KOs result in the presentation of a subset of noncanonical peptides that are presented in the wildtype.

The relative fraction of noncanonical peptides among all identified peptides differs significantly from the wildtype in TAP1, TAP2, and ERAP1 KOs (*p* = 0.05, *p* = 0.02, *p* = 0.05, respectively), with all three conditions exhibiting a decrease (Fig. 7*B*). These conditions directly impact the available peptide pool in the ER, suggesting that this change is primarily driven by altered peptide accessibility rather than modifications in peptide loading and editing.

The varying tendency of different HLA alleles to present noncanonical peptides has been previously reported (35). We also observed this pattern in the allele distribution of noncanonical peptides in our HAP1 panel (Fig. 7*C*). Interestingly, when we compared the fraction of noncanonical peptides to the fraction of canonical peptides bound by an allele, we found multiple significant differences between KOs and wildtype conditions (Fig. 7*C*). This shows that the changes in allele distribution upon APPM KOs changes differently for the canonical and noncanonical immunopeptidome.

## Discussion

In this study, we conducted a systematic, comparative, and in-depth immunopeptidomics analysis of PAKC, a panel of isogenic HAP1 cell lines, each carrying a single KO of an APPM protein. This panel is a validated collection of cell line models of APPM deficiency. Our in-depth analysis of these KOs revealed detailed ramifications that the silencing of APPM genes has. For each of the investigated KOs, direct impacts on the immunopeptidome were measurable. The effects ranged from minor reductions in immunopeptidome diversity (CALR) to complete obliteration of the immunopeptidome (B2M). The individual functions of each APPM proteins were reflected in multiple dimensions of the immunopeptidome, such as presentation capacity, antigen length preference, binding affinity, sampling localization, and allele dependency.

Regarding the canonical immunopeptidome, a principal phenomenon we observed is that knocking-out an APPM component led to the presentation of a subset of peptides that were also presented on the wildtype. This rule was consistent except for a few exceptions like TAP2, where the overlap with the wildtype was lower and mildly deviated from the wildtype immunopeptidome. Similarly, most source proteins that were found presented in KOs were also detected in wildtype samples. This indicates that a compromised APPM leads to compromised access to the pool of presentable peptides, resulting in decreased peptide diversity. Remarkably, despite knocking-out APPM components that are deemed crucial for presentation like TAPBP (36) and GANAB (37), it did not lead to the absence of presentation, which suggest a degree of redundancy within the APPM. This redundancy could arise from overlapping functions among different components or compensatory mechanisms that mitigate the loss of individual proteins. For example, while TAP1/TAP2 are critical for peptide translocation into the ER, other components involved in peptide loading and editing—such as TAPBP, PDIA3, and CALR—may have partially redundant roles, allowing for some level of functional compensation. In addition, compensation through chaperone networks or regulatory feedback loops could play a role in fine-tuning antigen processing and presentation, providing additional stability despite the loss of specific components (38).

Among our most remarkable findings are the changes in HLA allele–specific contributions to the immunopeptidome in the absence of TAPBP, TAP1, or TAP2. TAPBP plays a crucial role in peptide editing, a process that selects only the highest-affinity peptides to occupy the HLA groove before the HLA-I complex is transported to the cell surface. Our results clearly show the differential dependency of HLA alleles on different APPM players, and how their absence reshapes immunopeptidome composition in an HLA allele–dependent fashion. Of note, several HLA-I alleles are strongly linked with pathological conditions. For example, HLA-B alleles are known to have strong influences on acquired immunodeficiency syndrome progression, where several HLA-B alleles, such as HLA-B∗57 and HLA-B∗27, are protective in HIV infections, whereas other alleles, such as HLA-B∗35, are linked to rapid progression (39). Another example of HLA–disease associations is ankylosing spondylitis, which is associated with specific HLA-B∗27 variants (40). Furthermore, polyfunctional CD8 T-cell responses against HIV-1, cytomegalovirus, Epstein–Barr virus, and influenza were predominantly driven by virus epitopes restricted by HLA-B alleles (41), suggesting that allelic shifts because of APPM defects could impact the overall immunogenic potential of a sample. Further analyses across different HLA allotypes are needed to better understand the extent and implications for immune recognition of these shifts in allele distribution while factoring in changes in overall presentation levels.

We further elaborate on previous observations of varying degrees of TAP dependency by HLA allele (42). Individually knocking out either of the two TAP subunits resulted in varying degrees of impact on the immunopeptidome. For example, while the reduction in immunopeptidome diversity was significant in TAP1 KOs, it was even more pronounced in TAP2 KOs. A possible explanation for this is the capability of TAP2 to form homodimers. Upon knocking-out TAP1, TAP2 homodimers can substitute structurally for functional TAPs regarding their interaction with HLA (26). Despite lack of evidence for functional peptide transport by TAP2 homodimers, the interaction with HLA might suffice to support the PLC's ability to conduct peptide loading and editing for TAP-independent ligands. Similarly, the structural similarity, implying functional redundance, of CALR and CANX could explain why the immunopeptidome remained largely intact when either CALR or CANX was knocked-out. CANX and CALR are homologous, apart from CANX bearing a transmembranal domain (43).

One remarkable KO condition is ERAP1, in which the immunopeptide diversity dropped by more than half, indicating the significance of ERAP1 for the APPM. This is all the more relevant as ERAP1 inhibitors become increasingly relevant compounds of interest for cancer therapy research and development (44). This includes compounds already being tested in clinical trials (GRWD5769). Understanding the systemic effects of ERAP1 malfunction will prove valuable in assessing risks and benefits of future drugs.

The noncanonical immunopeptidome is of major interest to the cancer research community because a high fraction of cancer-specific immunopeptides originate from noncanonical sources and are shared between individuals (45, 46). In addition to previously reported findings that noncanonical peptides are presented at different rates by different HLA-I alleles (35), we shed light on changes of the noncanonical immunopeptidome upon APPM perturbation. APPM KOs present a subset of the immunopeptidome of the wildtype, for canonical and noncanonical immunopeptides alike. Hereby, the fraction of presented peptides that is noncanonical remains mostly the same, though noncanonical peptides depend more strongly on TAP translocation as well as trimming by ERAP1. We also show that the allele distribution of canonical and noncanonical immunopeptides can differ and react differently when the APPM is perturbed. Given the relatively low number of identified noncanonical immunopeptides, further research is needed to confirm this finding regarding changes in allele distribution within the noncanonical immunopeptidome.

Defects in the APPM are clinically relevant in different diseases. CALR defects, for instance, are associated with preneoplastic myeloproliferation in cancer (47). In colorectal cancer, TAP expression does not only closely correlate with HLA-I antigen expression but loss of TAP also correlates with decreased inflammatory responses by CD8^+^ tumor-infiltrating lymphocytes in the tumor (48). Under the light of our results and in agreement with previous studies (49), decreased inflammatory responses CD8^+^ tumor-infiltrating lymphocytes are expected upon TAP deficiency, considering the immense reduced immunopeptidome diversity. These examples showcase the relevance of understanding APPM mechanics.

Overall, this work represents the first systematic analysis of how the absence of individual APPM components change the immunopeptidome. Given the substantial variability in the effects of KOs on different HLAs, which exhibit varying degrees of reliance on the APPMs, it is crucial to investigate a broadeer range of HLAs under similar conditions. This approach could facilitate the creation of predictive tools capable of prioritizing HLA-bound peptides likely to be presented when presentation defects occur, such as in cancer and viral infections. Furthermore, such strategies could eventually help develop predictive methods to identify presentation defects, such as chromosomal aberrations combined with mutations in APPM key proteins, directly from bulk immunopeptidome samples.

## Data availability

DDA and DIA files of immunopeptidomics and proteomics experiments as well as the FASTA, library.tsv, report.tsv, and psm.tsv files were deposited on the ProteomeXchange Consortium *via* the PRIDE repository (50), dataset identifier PXD056426.

## Supplemental data

This article contains supplemental data (figures and tables).

## Conflict of interest

R. S. currently works at Neogene Therapeutics, a member of the AstraZeneca Group. All other authors declare no competing interests.

## Supplemental Data

Sup fig 1

Sup fig 2

Sup fig 3

Sup fig 4

Suppl-table1

Suppl-table2

Suppl-table3

Suppl-table4
