# Supplementary figures and images for: Deleterious KOs in the HLA Class I Antigen Processing and Presentation Machinery Induce Distinct Changes in the Immunopeptidome
Source: Mol Cell Proteomics. 2025 Mar 18;24(5):100951. doi: 10.1016/j.mcpro.2025.100951 (PMC12090245; doi:10.1016/j.mcpro.2025.100951)

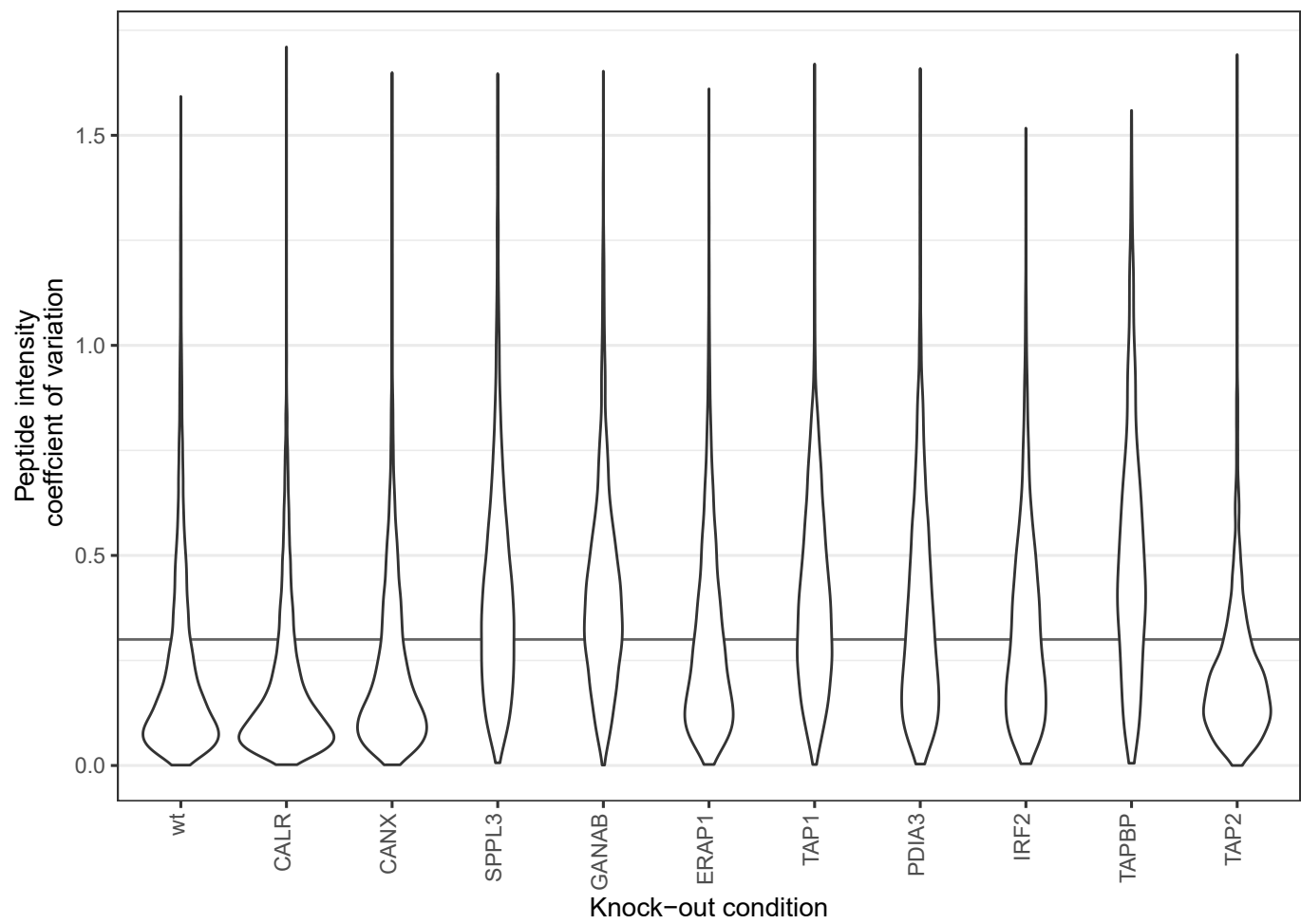

Supplement: Sup fig 1 [file mmc1.pdf]

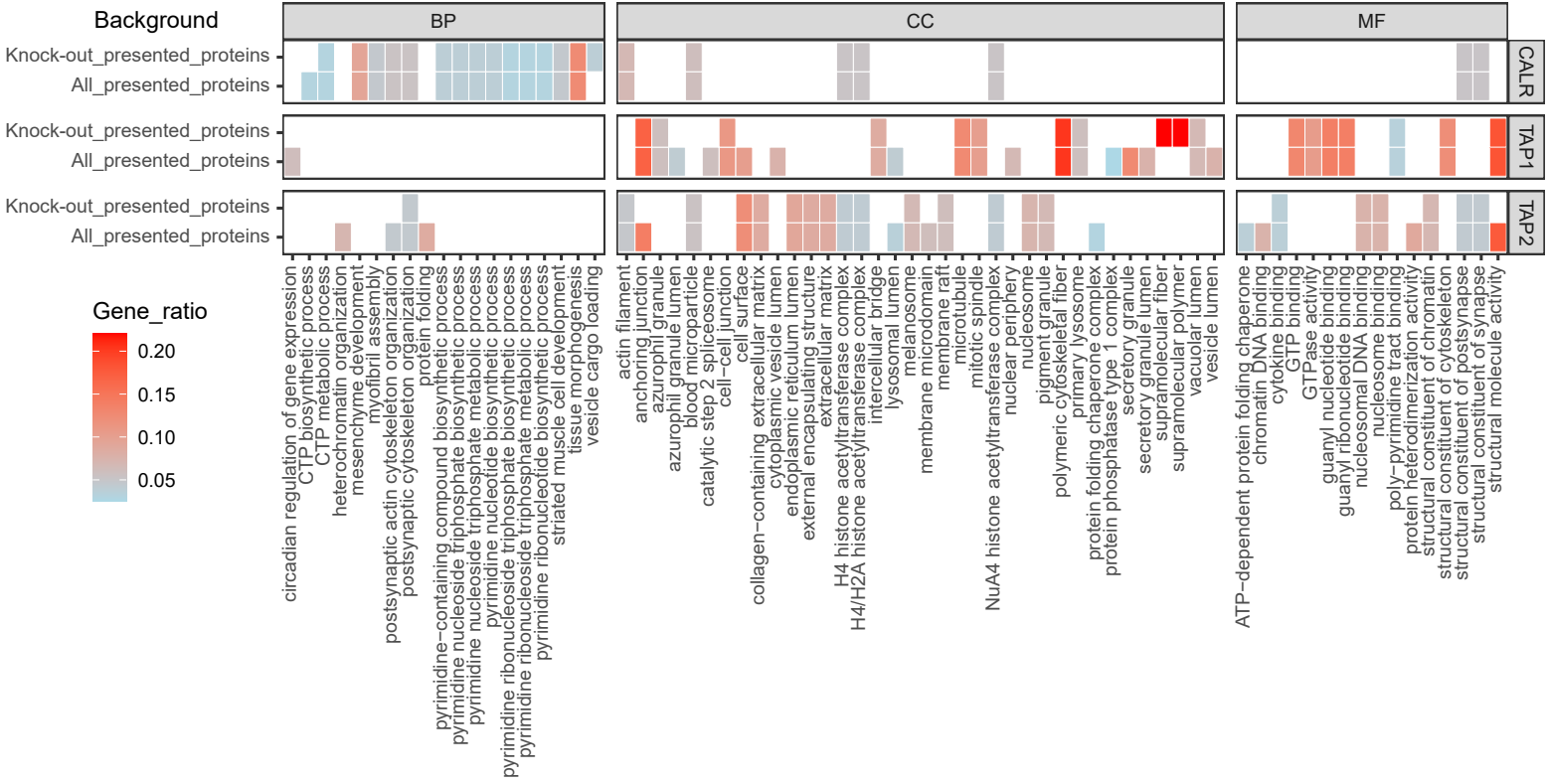

Supplement: Sup fig 2 [file mmc2.pdf]

A

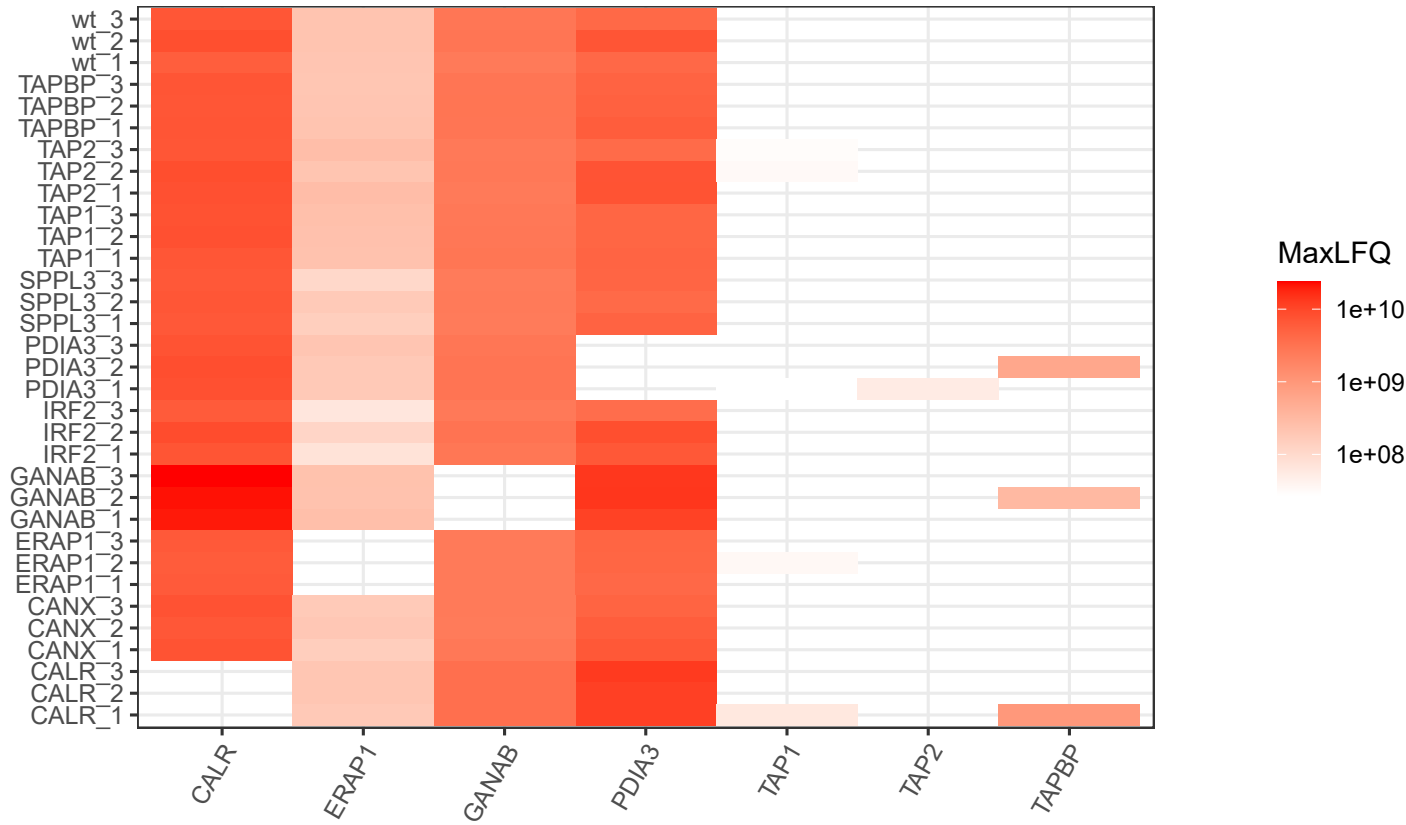

B

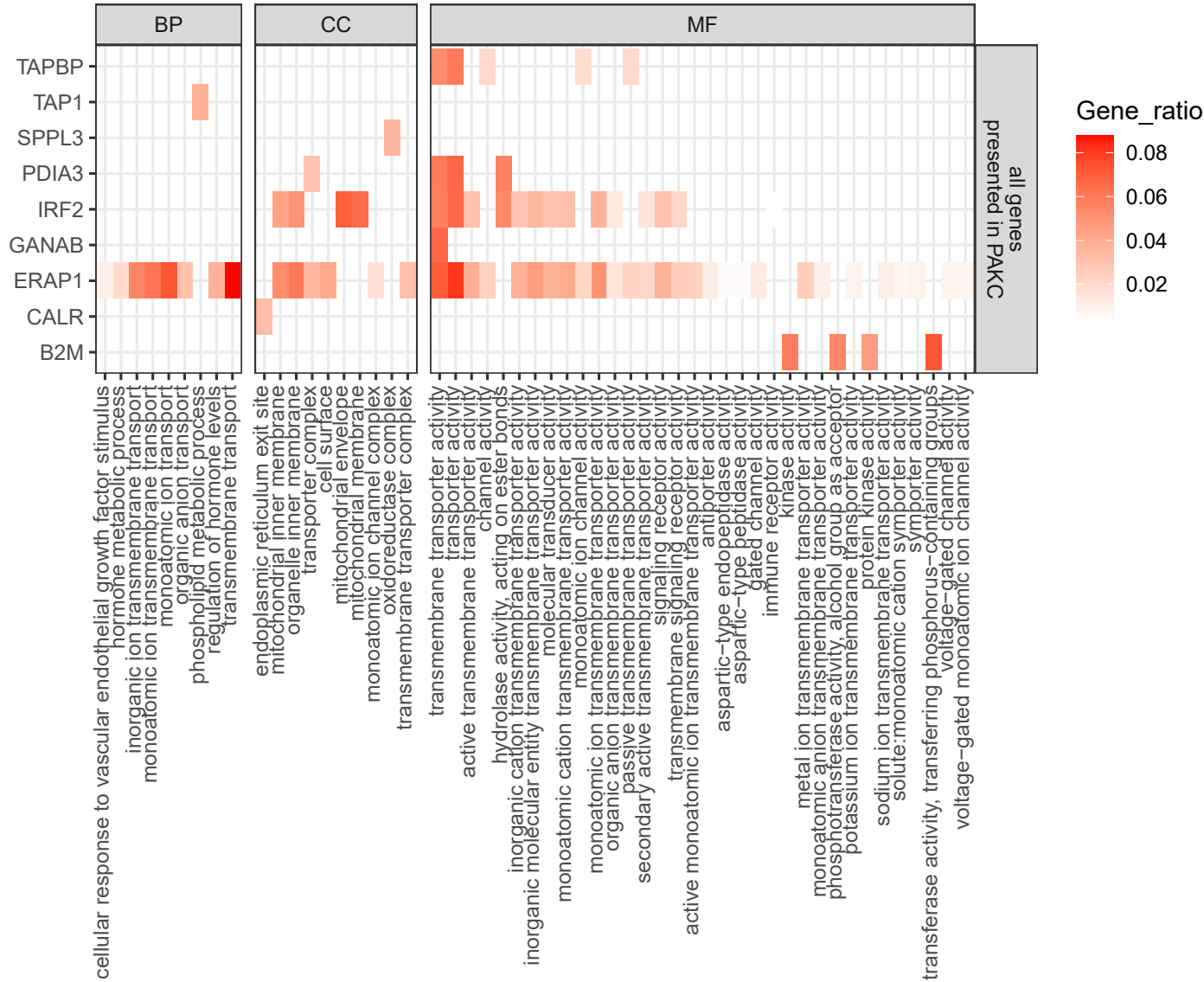

Supplement: Sup fig 3 [file mmc3.pdf]

A

Protein expression

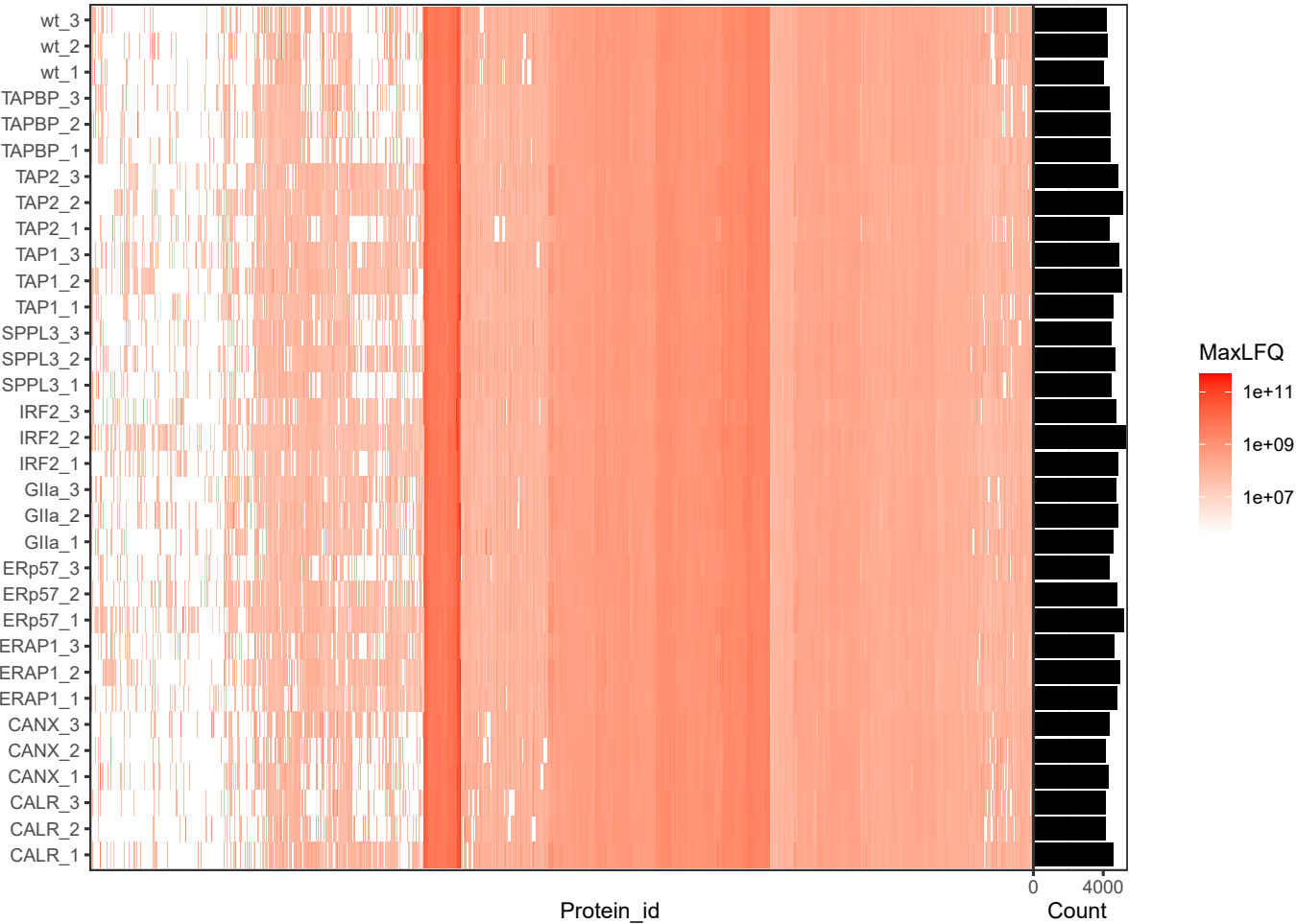

B

Protein sampling density

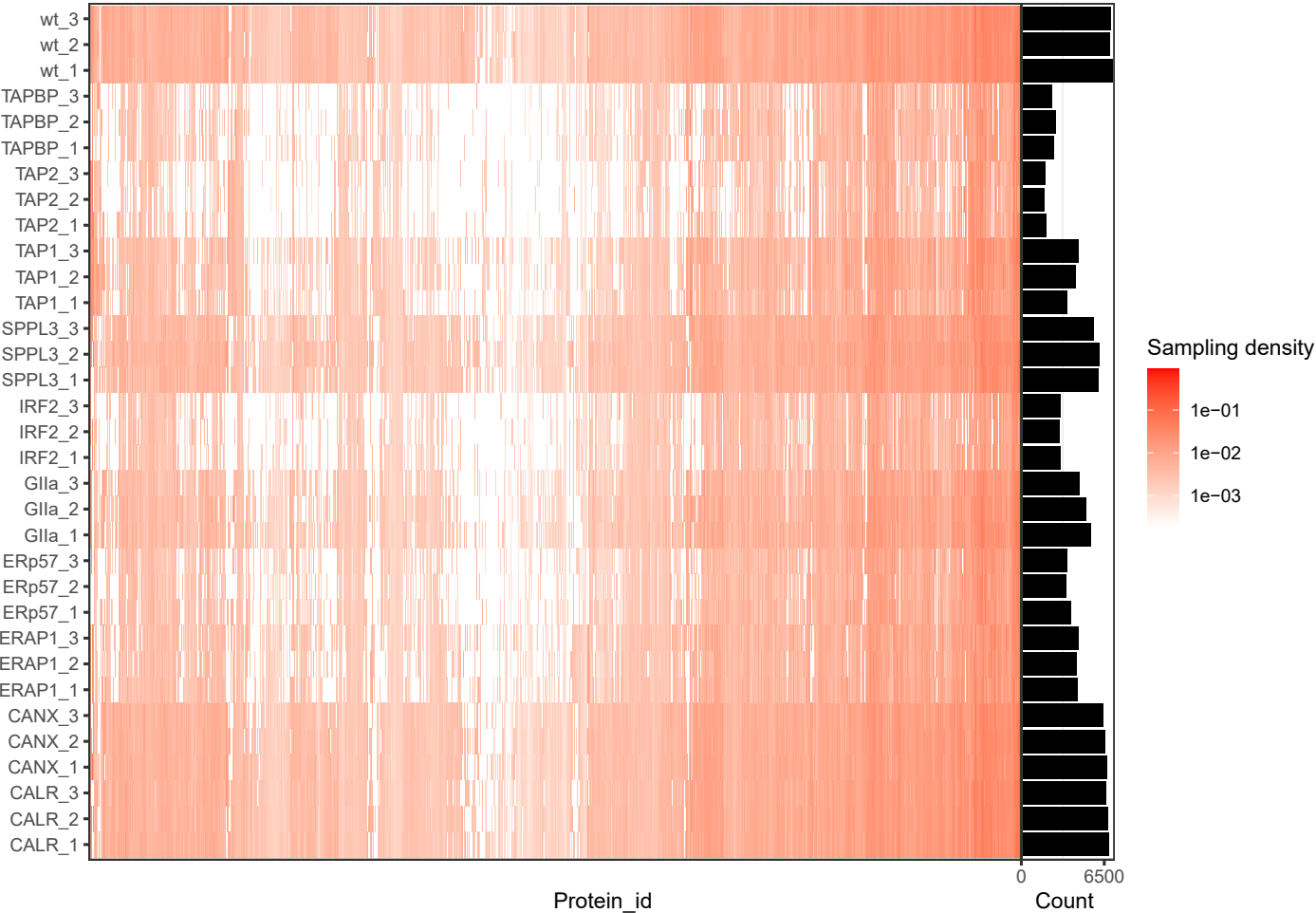

Supplement: Sup fig 4 [file mmc4.pdf]
